# Cyclopiazonic Acid-Induced Ca^2+^ Store Depletion Initiates Endothelium-Dependent Hyperpolarization-Mediated Vasorelaxation of Mesenteric Arteries in Healthy and Colitis Mice

**DOI:** 10.3389/fphys.2021.639857

**Published:** 2021-03-09

**Authors:** Lu Yun Zhang, Xiong Ying Chen, Hui Dong, Feng Xu

**Affiliations:** ^1^Department of Pediatric Intensive Care Unit, Children’s Hospital of Chongqing Medical University, National Clinical Research Center for Child Health and Disorders, Ministry of Education Key Laboratory of Child Development and Disorders, Chongqing Key Laboratory of Pediatrics, Chongqing, China; ^2^Department of Gastroenterology, Xinqiao Hospital, Army Medical University, Chongqing, China

**Keywords:** store-operated calcium entry, endothelium-dependent hyperpolarization, cyclopiazonic acid, colitis, mesenteric arteries

## Abstract

**Purposes**: Since the role of store-operated calcium entry (SOCE) in endothelium-dependent hyperpolarization (EDH)-mediated vasorelaxation of mesenteric arteries in health and colitis is not fully understood, cyclopiazonic acid (CPA), a specific inhibitor of the sarco(endo) plasmic reticulum calcium-ATPases (SERCA), was used as a SOCE activator to investigate its role in normal mice and its alteration in colitis mice.

**Methods**: The changes in Ca^2+^ signaling in vascular endothelial cells (VEC) were examined by single cell Ca^2+^ imaging and tension of mesenteric arteries in response to CPA were examined using Danish DMT520A microvascular measuring system.

**Results**: CPA activated the SOCE through depletion of the endoplasmic reticulum (ER) Ca^2+^ in endothelial cells. CPA had a concentration-dependent vasorelaxing effect in endothelium-intact mesenteric arteries, which was lost after endothelial removal. Both nitric oxide (NO) and prostacyclin (PGI_2_) inhibitors did not affect CPA-induced vasorelaxation; however, after both NO and PGI_2_ were inhibited, K_Ca_ channel blocker [10 mM tetraethylammonium chloride (TEA)] inhibited CPA-induced vasorelaxation while K_Ca_ channel activator (0.3 μM SKA-31) promoted it. Two SOCE blockers [30 μM SKF96365 and 100 μM flufenamic acid (FFA)], and an Orai channel blocker (30 μM GSK-7975A) inhibited this vasorelaxation. The inhibition of both Na^+^/K^+^-ATPase (NKA) and Na^+^/Ca^2+^-exchange (NCX) also inhibited CPA-induced vasorelaxation. Finally, the CPA involved in EDH-induced vasorelaxation by the depletion of ER Ca^2+^ of mesenteric arteries was impaired in colitis mice.

**Conclusion**: Depletion of ER Ca^2+^ by CPA induces a vasorelaxation of mesenteric arteries that is mediated through EDH mechanism and invokes the activation of SOCE. The CPA-induced endothelium-dependent dilation is impaired in colitis which may limit blood perfusion to the intestinal mucosa.

## Introduction

Ca^2+^ as an important second messenger plays a critical role in the regulation of cell function and participates in various human physiological processes. In the normal resting state, there is a fine regulation of the cellular Ca^2+^ levels, such that the free intracellular Ca^2+^ ([Ca^2+^]_i_) is much lower than the extracellular Ca^2+^. When cells are stimulated, the rapid influx of extracellular Ca^2+^ increases the concentration of [Ca^2+^]_i_, which is an important signal that triggers many physiological activities in the cells (Garland et al., 2017). Putney first proposed the concept of the store-operated calcium entry (SOCE), a physiological phenomenon that the depletion of Ca^2+^ store in the endoplasmic reticulum (ER) activates the influx of extracellular Ca^2+^ (Putney, 1990). The molecular mechanism of the SOCE is comprised of the STIM protein of the endoplasmic reticulum membrane and the Orai protein family of the cell membrane. The STIM protein senses a decrease in the ER Ca^2+^, and activates the Orai protein located on the cell membrane through protein-protein interactions, thereby causing the influx of extracellular Ca^2+^ (Liou et al., 2005; Zhang et al., 2006). Under physiological conditions, the SOCE can be activated by GPCR/PLC/IP_3_-mediated ER Ca^2+^ release (Taylor, 2006).

Vascular endothelial cells (VEC) play an important role in regulating vascular function by producing three relaxing signals: nitric oxide (NO; Kruse et al., 1994; Zuccolo et al., 2016), prostacyclin (PGI_2_; Asai et al., 2009), and endothelium-dependent hyperpolarization (EDH; Félétou and Vanhoutte, 2007, 2009). EDH plays a major role in regulating the relaxation of fine resistance blood vessels (Garland et al., 1995; Crane et al., 2003), while NO and PGI_2_ in that of large blood vessels (Guo et al., 2018). Although the nature of EDH has not been fully identified, the endothelial Ca^2+^-activated K_Ca_ channels are generally accepted as irreplaceable components of EDH signal (Cocks et al., 1988; Garland and Dora, 2017). In VEC, the SOCE has an important effect on the fine regulation of [Ca^2+^]_i_. Physiologically, the SOCE was shown to mainly involve in the acetylcholine (ACh)/NO-induced vasorelaxation (Lin et al., 2000; Dedkova and Blatter, 2002). Edwards stated that CPA contributes to EDH-induced vasorelaxation by the depletion of ER Ca^2+^ (Edwards et al., 2008), however, the role of SOCE in this phenomenon is not well established.

Inflammatory bowel disease (IBD) is a group of chronic inflammatory diseases, including Crohn’s disease (CD) and ulcerative colitis (UC), and there is no effective treatment for them currently. Numerous studies on IBD focused on intestinal mucosal barrier damage and immune dysfunction, while only a few investigated the involvement of mesenteric circulation in the pathogenesis of IBD. Mesenteric arteries in IBD patients have weakened vasorelaxation in response to ACh, resulting in reduced blood flow in the inflamed area (Hatoum et al., 2003a; Hatoum and Binion, 2005). This dysfunction in the mesenteric vasorelaxation affects the blood supply in the intestinal mucosa, thereby promoting the progression of IBD. Understanding the role of intestinal blood circulation in IBD may have theoretical significance and clinical implication; however, it has not been explored at present whether the CPA/SOCE/EDH of mesenteric endothelial cells is altered in the progression of IBD. Therefore, in this study, we aimed to explore the regulatory mechanisms of CPA/SOCE/EDH action on mesenteric arteries in healthy and colitis mice to provide new potential targets for the prevention/treatment of colitis.

## Materials and Methods

### Animals

The animal studies were approved by the Ethics Committee of Chongqing Medical University, Chongqing, China. Experiments were conducted on male C57BL/6 mice (6–12 weeks-old; 20–25 g), which were purchased from Chongqing Tengxin Biotechnology Co. Ltd., Chongqing, China. The mice were housed in polypropylene plastic cages with unlimited access to tap water, with up to a maximum of five animals per cage, in a temperature-controlled room with a 12/12-h light/dark cycle. The mice were anesthetized using 100% CO_2_ and euthanized through cervical dislocation. Before each experiment, the mice were deprived of food and water for at least 1 h. For all animal experiments, only male mice were used to minimize possible variations owing to the sex of the animal.

Animal studies were conducted in accordance to the ARRIVE guidelines (Kilkenny et al., 2010; McGrath and Lilley, 2015). The protocols were in compliance with the Army Military Medical University Committee on Investigations Involving Animal Subjects. All animal care and experimental studies were conducted in accordance with the guidelines of the Animal Ethical Committee of Chongqing Medical University and the Guide for the Care and Use of Laboratory Animals published by the United States National Institutes of Health (NIH Publication No. 85–23, revised 1996).

### Cell Culture

Human umbilical vein endothelial cells (HUVECs; American Type Culture Collection, Manassas, VA, United States) were cultured in RPMI-1640 (Hyclone, Waltham, MA, United States) containing 10% fetal bovine serum (FBS; Gibco, Gaithersburg, MD, United States) and 1% Penicillin–Streptomycin (Beyotime Biotechnology, China) at 37°C under 5% CO_2_ and saturated humidity. Cells were plated on glass coverslips about 24 h before experiments.

### Measurement of [Ca^2+^]_i_ by Digital Ca^2+^ Imaging

Ca^2+^ imaging experiments were performed as previously described (Wan et al., 2017). Cells grown on coverslips were loaded with 5 μM Fura-2/AM in physiological salt solution (PSS), described below, at 37°C for 60 min and then washed for 20 min. Thereafter, the coverslips with HUVEC were mounted in a perfusion chamber on a Nikon microscope stage (Nikon Corp, Tokyo, Japan). The ratio of Fura-2/AM fluorescence with excitation at 340 or 380 nm (F_340/380_) was followed over time and captured using an intensified charge-coupled device camera (ICCD200) and a MetaFluor imaging system (Universal Imaging Corp, Downingtown, PA). In our study, fluorescence ratios from single cells were recorded at 3 s intervals, so the sampling rate used in our study was 1/3 fs (Hz). The Ca^2+^ imaging system was calibrated using Titration Calibration *in situ* according to MetaFluor Online Help. F340/380 ratio measurements were performed and imaged every 3 s. The PSS used in digital Ca^2+^ measurement contained the following: 140 mM Na^+^, 5 mM K^+^, 2 mM Ca^2+^, 147 mM Cl^−^, 10 mM Hepes, and 10 mM glucose (pH 7.4). For the Ca^2+^-free PSS, Ca^2+^ was omitted, but 0.5 mM EGTA was added. The osmolality for all solutions was ~300 mosmol/kg of H_2_O.

### Myograph Experiments

The mesenteric artery is a recognized microvascular model, which is often used to study the physiological and pathological mechanism of resistance vessels; therefore, we used mesenteric arteries as the representative model in this study. The C57BL/6 mice were sacrificed, abdomen was fully exposed, and the mesangial intestinal tube was quickly removed and placed in pre-cooled Krebs–Henseleit solution. Krebs–Henseleit solution contained 118 mM NaCl, 4.7 mM KCl, 1.18 mM MgSO_4_, 25 mM NaHCO_3_, 1.2 mM KH_2_PO_4_, 1.6 mM CaCl_2_, and 11.1 mM D-glucose. The fat and connective tissues around the blood vessels were carefully removed under a microscope. The mesenteric arteries (100–150 μm of diameter, 2-mm segments in length) were obtained from the second-order branch of the superior mesenteric artery and placed in Krebs solution. Two tungsten wires (each 40 μm in diameter) were passed through the mesenteric arteries, which were fixed to jaws of the Mulvany-style wire myograph (Model 520A, DMT, Aarhus, Denmark) for functional assessment. Isometric tension changes were recorded using a Powerlab analytical system (AD Instruments, Colorado Springs, CO, United States). The chamber bath contained 5 ml K-H solution, the bath temperature was maintained at 37°C, and a mixture of 95% O_2_ + 5% CO_2_ was injected and maintained at a pH of ~7.4. One side of the tungsten wire was connected to the tension transducer, and the other side was connected to the blood vessel fine-tuning device.

The vascular endothelium was removed by rubbing the luminal surface of the mesenteric arteries for several times using human hair. Successful endothelial denudation was verified by a lack (≤10%) of vasorelaxation response to CCh (100 μM). The experiments were performed after the successful removal of the vascular endothelium.

### Concentration-Response Curve

Cumulative concentration-response curve (CRC) to cyclopiazonic acid (CPA, 4–12 μM) was performed in norepinephrine (NE, 10 μM)‐ or KCl (80 mM)-pre-constricted arteries. Since the *in situ* blood vessels are under a transmural pressure by neurohumoral regulation, the isolated blood vessels must be standardized to meet physiological tension in Krebs–Henseleit solution for at least 20 min. After standardization, the tension was equivalent to 0.9 times the blood vessel diameter under 100 mmHg pressure. The blood vessels were first incubated with different drugs for 20 min, and then the cumulative CRC to CPA (4, 6, 8, 10, and 12 μM) were performed in NE (5 μM)‐ or KCl (80 mM)-preconstricted arteries.

To understand the mechanism underlying CPA-induced vasorelaxation, arterial rings were treated for 20 min with the following activators and inhibitors: indomethacin (INDO, 10 μM, inhibitor of cyclooxygenase, COX), Nω-nitro-L-arginine (L-NNA, 100 μM, inhibitor of nitric oxide synthase, NOS), ouabain (100 μM, inhibitor of Na^+^/K^+^-ATPase, NKA), SN-6 (10 μM, inhibitor of Na^+^/Ca^2+^ exchanger, NCX), GSK-7975A (30 μM, blocker of SOCE), flufenamic acid (FFA; 100 μM, blocker of SOCE, FFA), SKF96365 (30 μM, blocker of SOCE), tetraethylammonium chloride (TEA, 10 mM, blocker of Ca^2+^-activated K^+^ channels, K_Ca_), and SKA-31 (0.3 μM, activator of Ca^2+^-activated K^+^ channels, K_Ca_).

### Dextran Sulfate Sodium-Induced Colitis Mouse Model

Dextran sulfate sodium (DSS)-induced colitis mouse is a commonly used animal model for studying colitis (Okayasu et al., 1990; Meir et al., 2019). Twelve healthy male C57 mice (6–8 weeks, 17–23 g) were randomly divided into two groups. The control group was fed drinking water, and the test group was fed water with 2.5% DSS, for 7 days (labeled as days 1–7). Mice were monitored daily for body weight, rectal bleeding, and water consumption. After 4 days, the mice in the test group developed bloody stools and started losing weight. On day 7, the mice were anesthetized using 100% CO_2_ and sacrificed through cervical dislocation. The lengths of the colons of the mice in the two groups were measured.

### Materials

Cyclopiazonic acid, L-NNA, carbachol (CCH), TEA, ACh, and INDO were purchased from Sigma. Ouabain was purchased from ApexBio. SKA-31, SKF96365, and GSK-7975A were purchased from MedChemExpress. SN-6 was purchased from Tocris. The most of the reagents were dissolved in DMSO at final concentration of less than 0.1%, which did not alter vascular activities in the experiments.

### Data and Statistics Analysis

All results are expressed as mean ± SE, with *n* representing the number of animals, and no data points were excluded from the analysis in any of the results. Furthermore, the sample sizes of animal experiments have taken the 3Rs principles into consideration (Kilkenny et al., 2010). All results are means ± SE with *n* represents the number of animals and *n* ≥ 6 in each group of experiments. In cell experiments (Ca^2+^ imaging), *n* represented the number of cells. For all studies, animals were randomly assigned to different experimental groups. GraphPad Software 6.0 (San Diego, CA) was used to determine the cumulative CRC, maximal relaxation (R_max_), and the concentration for 50% maximal effect (EC_50_). The statistical significance of differences in the means of experimental groups was determined using unpaired, two-tailed Student’s *t*-test for two groups or one-way ANOVA. Dunnett’s post or *post hoc* tests were performed only if *F* achieved *p* < 0.05 (GraphPad Prism 7.0, GraphPad Software, Inc., RRID: SCR_002798) for multiple groups. *p* < 0.05 was considered statistically significant.

## Results

### CPA Activated the SOCE in Vascular Endothelial Cells

**Figure 1:**
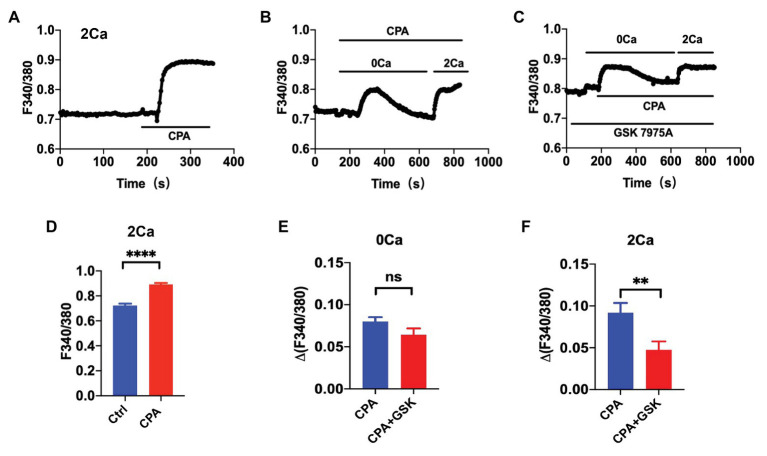
The cyclopiazonic acid (CPA)-induced store-operated calcium entry (SOCE) in single vascular endothelial cells. **(A)** Summary data showing the time courses of CPA (10 μM)-induced Ca^2+^ signaling in Ca^2+^-containing solution (2Ca, *n* = 17 cells). **(B)** Summary data showing the time courses of CPA-induced Ca^2+^ signaling in Ca^2+^-free solution (0Ca) and after restoration of extracellular Ca^2+^ (2Ca, *n* = 16 cells). **(C)** Summary data showing the time courses of CPA-induced Ca^2+^ signaling in 0Ca and after restoration of 2Ca in the presence of GSK-7975A (30 μM, *n* = 16 cells). **(D)** Summary data showing CPA-induced Ca^2+^ signaling in Ca^2+^-containing solution (2Ca, *n* = 17 cells). **(E)** Summary data showing the delta Ca^2+^ signaling induced by CPA in Ca^2+^-free solution (0Ca, *n* = 16 cells) in the absence or the presence of GSK-7975A (30 μM). **(F)** Summary data showing the delta Ca^2+^ signaling induced by CPA after restoration of 2Ca in the absence or the presence of GSK-7975A (30 μM, *n* = 16 cells). Data were shown as means ± SEM. ^**^p < 0.01, ^****^p < 0.0001, and ns, no significance.

Cyclopiazonic acid is a well-known specific inhibitor of the sarco(endo) plasmic reticulum calcium-ATPases (SERCA). The SERCA inhibition can deplete the ER Ca^2+^ to presumably activate the SOCE. We examined the CPA-induced SOCE in HUVEC. First, after basal [Ca^2+^]_i_ was stable in normal PSS containing 2 mM extracellular Ca^2+^, application of CPA (10 μM) induced a marked increase in [Ca^2+^]_i_ in HUVEC (Figure 1A). Second, in the absence of extracellular Ca^2+^ (0Ca PSS), CPA induced a transient increase in [Ca^2+^]_i_ due to Ca^2+^ release from the ER to the cytosol. When the store was depleted (i.e., when the [Ca^2+^]_cyt_ transients declined back to the basal level), restoration of extracellular Ca^2+^ to 2 mM (2Ca) induced a further increase in [Ca^2+^]_i_ due to Ca^2+^ entry through the SOCE (Figure 1B). Third, GSK-7975A (30 μM), a selective Orai blocker, did not affect the CPA-induced transient increase in [Ca^2+^]_i_ due to Ca^2+^ release from the ER, but significantly attenuated the further increase in [Ca^2+^]_i_ due to Ca^2+^ entry through the SOCE (Figure 1C). Figure 1D summarizes the CPA-induced [Ca^2+^]_i_ in HUVEC in 2Ca PSS, and Figures 1E,F summarize effect of GSK-7975A on the CPA-induced [Ca^2+^]_i_ in 0Ca PSS and after restoring 2Ca. These results indicate that CPA indeed activates the SOCE, and thus it can be reasonably used as a selective SOCE activator in VEC.

### CPA-Induced Endothelium-Dependent Vasorelaxation

**Figure 2:**
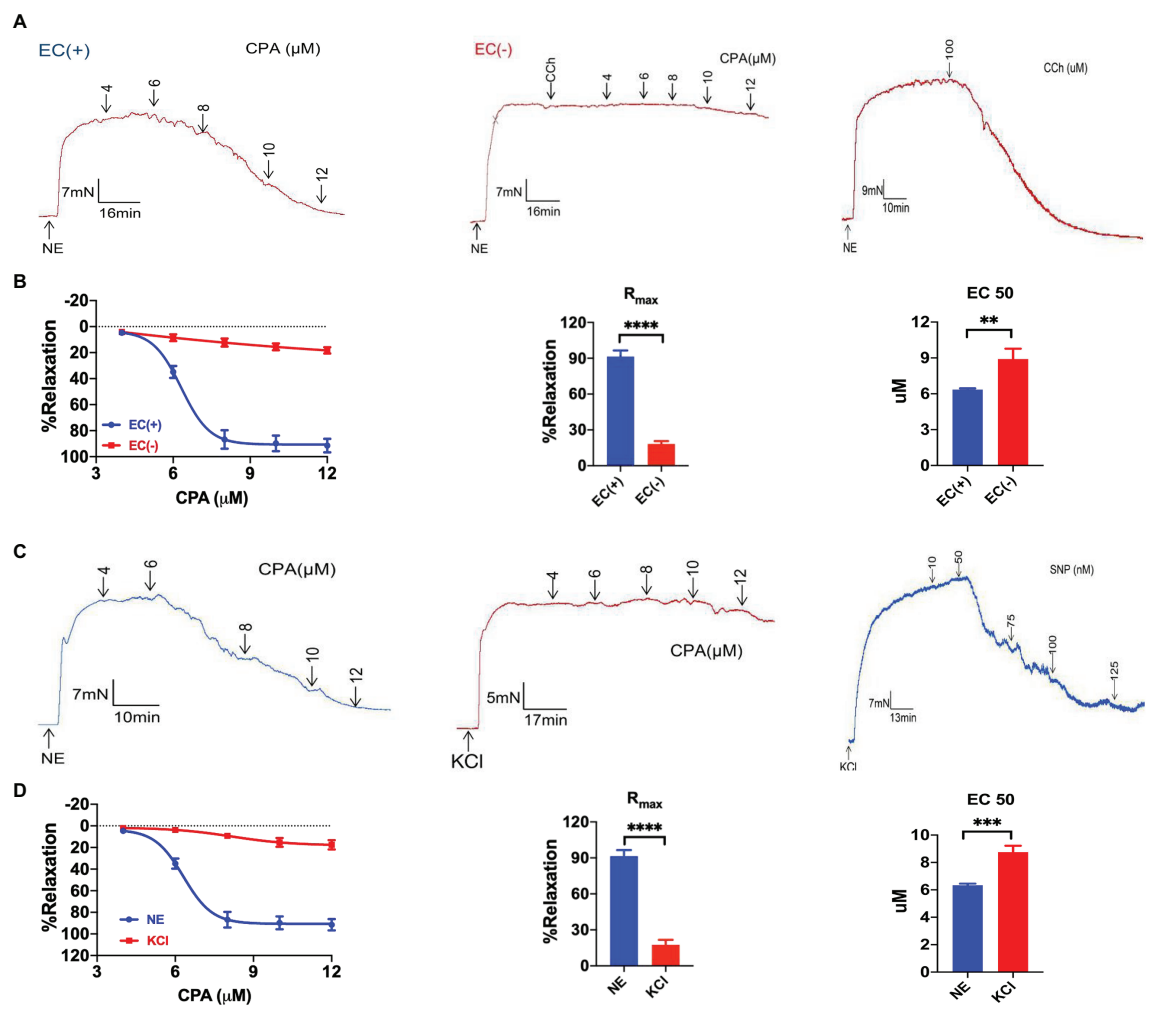
Cyclopiazonic acid-induced endothelium-dependent and extracellular K^+^-sensitive vasorelaxation of mesenteric arteries. **(A)** Representative tracings showing CPA or carbachol (CCh, 100 μM) induced endothelium-dependent vasorelaxation with intact endothelium (EC+, the left panel; the right panel) and the loss of vasorelaxation in response to CCh (100 μM) or CPA in endothelium-denuded arteries (EC−, the middle panel). **(B)** Summary data showing concentration-response curve (CRC), R_max_, and EC_50_ of CPA-induced vasorelaxation with intact endothelium (EC+, *n* = 6) or denuded endothelium (EC−, *n* = 6). **(C)** Representative tracings of CPA-induced concentration-dependent vasorelaxation in mesenteric arteries preconstricted with noradrenalin (5 μM NE, the left panel), and CPA or SNP concentration-dependent vasorelaxation in mesenteric arteries preconstricted with high extracellular K^+^ (80 mM KCl, the middle and the right panels). **(D)** Summary data showing the CRC, R_max_, and EC_50_ of CPA-induced vasorelaxation in mesenteric arteries preconstricted with norepinephrine (NE; *n* = 6) or KCl (*n* = 6). Data were expressed as percentage of NE‐ or KCl-induced vasoconstriction and shown as means ± SEM. ^**^p < 0.01, ^***^p < 0.001, ^****^p < 0.0001.

Next, we used CPA as a selective SOCE activator to examine whether CPA induced vasorelaxation. CPA at 4–12 μM induced marked vasorelaxation of the arteries pre-constricted using NE (5 μM) in a concentration-dependent manner (NE-vasoconstriction value 9.51 ± 1.26 mN, R_max_ 91.46 ± 5.09%, and EC_50_ 6.18 ± 0.07 μM; Figures 2A,B). To test if CPA-induced vasorelaxation was endothelium-dependent, we compared CPA-induced vasorelaxation between endothelium-intact and endothelium-denuded mesenteric arteries. In endothelium-denuded mesenteric arteries, which was confirmed using CCh (100 μM, R_max_ 6.32 ± 1.49%, and *n* = 6), CPA induced only 20% vasorelaxation (R_max_ 18.29 ± 2.33%, *n* = 6; Figures 2A,B). Therefore, CPA predominantly induced endothelium-dependent and concentration-dependent vasorelaxation.

### CPA-Induced Mesenteric Arterial Relaxation Through EDH

Vasorelaxation of mesenteric arteries plays a critical role in controlling blood flow perfusion in mesenteric circulation, which maintains normal mucosal barrier function in the intestine of healthy subjects (Aneman et al., 1997; Hatoum et al., 2003a). CPA induced marked vasorelaxation of the arteries pre-constricted using NE (5 μM) in a concentration-dependent manner, but induced only a marginal vasorelaxation of the arteries pre-constricted using high K^+^ (80 mM; Figures 2C,D). The CPA-induced CRC and R_max_ were much greater in the arteries pre-constricted using NE (R_max_ 91.46 ± 5.09%, *n* = 6) compared to those pre-constricted using high K^+^ (R_max_ 17.51 ± 4.17%, *n* = 6, *p* < 0.05; Figure 2D). Therefore, CPA induced much greater vasorelaxation of the arteries pre-constricted using NE than those pre-constricted using high K^+^, in a concentration-dependent manner, suggesting that K^+^ channels possibly participate in the CPA-induced vasorelaxation (Li et al., 2015).

**Figure 3:**
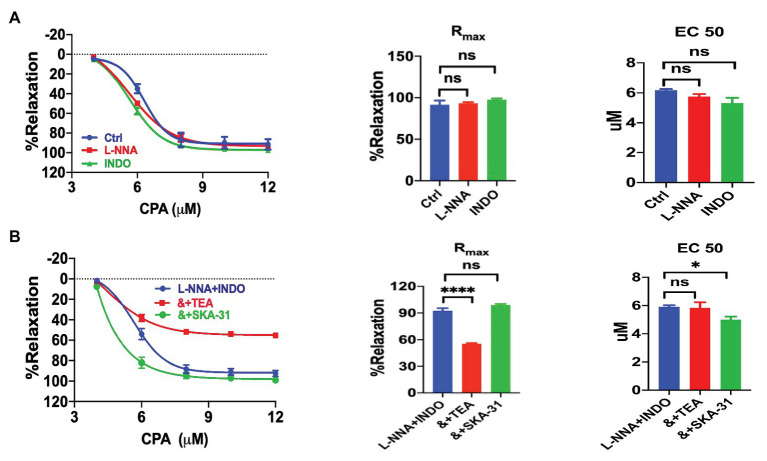
Cyclopiazonic acid induction of mesenteric arterial relaxation through endothelium-dependent hyperpolarization. **(A)** Summary data showing the CRC, R_max_, and EC_50_ of CPA-induced vasorelaxation in mesenteric arteries in the absence (control, *n* = 6) or the presence of either 100 μM Nω-nitro-L-arginine (L-NNA; *n* = 6) or 10 μM indomethacin (INDO; *n* = 6). **(B)** Summary data showing the CRC, R_max_, and EC_50_ of CPA-induced vasorelaxation in the presence of L-NNA + INDO (*n* = 6), L-NNA + INDO (&) + 0.3 μM SKA-31 (*n* = 6), or L-NNA + INDO (&) + 10 mM tetraethylammonium chloride (TEA; *n* = 6). Data were expressed as percentage of NE (5 μM)-induced vasoconstriction and shown as means ± SEM. ^*^p < 0.05, ^****^p < 0.0001, and ns, no significance.

We explored the underlying mechanisms of CPA-induced vasorelaxation. VEC are known to generate three different endothelium-derived relaxing factors: NO, PGI_2_, and EDH (Félétou and Vanhoutte, 2007, 2009). Neither NO inhibitor L-NNA (100 μM, *n* = 6, NE-vasoconstriction value 6.40 ± 0.62 mN) nor PGI_2_ inhibitor INDO (10 μM, *n* = 6, NE-vasoconstriction value 6.67 ± 0.55 mN) affected CPA-induced vasorelaxation (Figure 3A). Similarly, the combination of L-NNA and INDO did not affect the CPA-induced vasorelaxation (Figure 3B; R_max_ 92.55 ± 2.79%, EC_50_ 5.92 ± 0.10 μM, NE-vasoconstriction value 9.74 ± 0.86 mN), further supporting that both NO and PGI_2_ play minor roles in the vasorelaxation, while EDH may play a major role.

The CPA-induced vasorelaxation through EDH was selected for further analysis after L-NNA and INDO were applied to inhibit the endothelium-dependent vasorelaxation through NO and PGI_2_. A large portion of CPA-induced vasorelaxation was further attenuated by TEA (10 mM, R_max_ 55.31 ± 0.93%, EC_50_ 5.83 ± 0.40 μM, NE-vasoconstriction value 6.47 ± 1.12 mN), a blocker of K_Ca_ channels; but potentiated by SKA-31 (0.3 μM, NE-vasoconstriction value 6.92 ± 0.74 mN), a selective IK_Ca_ and SK_Ca_ channel activator that can in turn potentiate EDH-type arterial dilation (Sankaranarayanan et al., 2009). The inhibitory effect of TEA and the potentiation effect of SKA-31 on CPA-induced CRC, R_max_, and EC_50_ in the presence of L-NNA and INDO are summarized in Figure 3B. Taken together, CPA-induced vasorelaxation is mainly dependent on EDH.

### The EDH-Mediated Vasorelaxation Depended on the SOCE Mechanism

**Figure 4:**
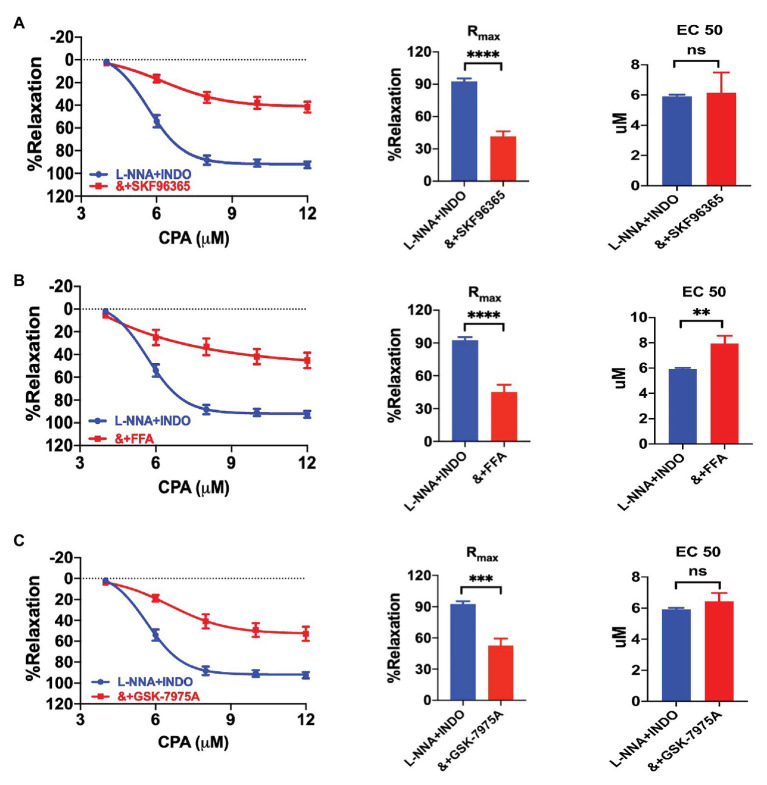
Cyclopiazonic acid induced vasorelaxation through the SOCE/endothelium-dependent hyperpolarization (EDH) mechanism. **(A)** Summary data showing the CRC, R_max_, and EC_50_ of CPA-induced vasorelaxation in the presence of either L-NNA + INDO (*n* = 6) or L-NNA + INDO (&) + 30 μM SFK96365 (*n* = 6). **(B)** Summary data showing the CRC, R_max_, and EC_50_ of CPA-induced vasorelaxation in the presence of either L-NNA + INDO (*n* = 6) or L-NNA + INDO (&) + 100 μM flufenamic (FFA, *n* = 6). **(C)** Summary data showing the CRC, R_max_, and EC_50_ of CPA-induced vasorelaxation in the presence of either L-NNA + INDO (*n* = 6) or L-NNA + INDO (&) + 30 μM GSK-7975A (*n* = 6). Data were expressed as percentage of NE (5 μM)-induced vasoconstriction and shown as means ± SEM. ^**^p < 0.01, ^***^p < 0.001, ^****^p < 0.0001, and ns, no significance.

We further investigated if CPA-induced vasorelaxation through EDH depends on the SOCE mechanism. Indeed, CPA-induced CRC was significantly attenuated by selective SOCE blockers SKF96365 (30 μM), FFA (100 μM), and Orai blocker GSK-7975A (30 μM), respectively, in the presence of L-NNA + INDO (Figures 4A–C). As shown in Figures 4A–C, they significantly reduced CPA-induced R_max_. Compared with L-NNA + INDO (R_max_ 92.55 ± 2.79%), the CPA-induced R_max_ were significantly reduced by SKF963659 (R_max_ 41.65 ± 4.64%, NE-vasoconstriction value 6.88 ± 0.70 mN, *p* < 0.0001), FFA (R_max_ 45.13 ± 6.65%, NE-vasoconstriction value 8.62 ± 0.70 mN, *p* < 0.0001), and GSK-7975A (R_max_ 52.84 ± 6.62%, NE-vasoconstriction value 7.20 ± 0.99 mN, *p* < 0.0001). In summary, CPA induces an endothelium-dependent vasorelaxation through the SOCE/EDH mechanism.

### Na^+^-K^+^ ATPase in the SOCE/EDH-Mediated Vasorelaxation

**Figure 5:**
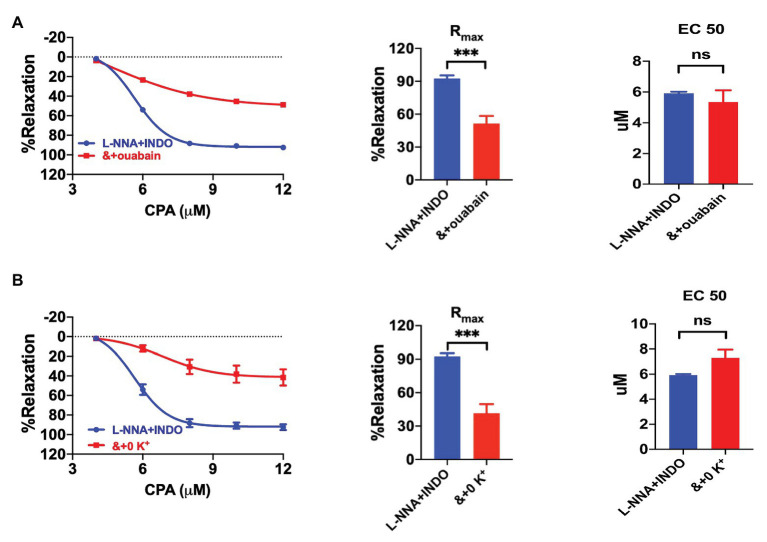
The involvement of Na^+^-K^+^-ATPase (NKA) in the EDH-mediated vasorelaxation of mesenteric arteries. **(A)** Summary data showing the CRC, R_max_, and EC_50_ of CPA-induced vasorelaxation in the presence of either L-NNA + INDO (*n* = 6) or L-NNA + INDO (&) + 100 μM ouabain (*n* = 6). **(B)** Summary data showing the CRC, R_max_, and EC_50_ of CPA-induced vasorelaxation in the presence of either L-NNA + INDO (*n* = 6) or L-NNA + INDO (&) + 0 K^+^ (*n* = 6). Data were expressed as percentage of NE (5 μM)-induced vasoconstrictions and shown as means ± SEM. ^***^p < 0.001, and ns, no significance.

Since SK_Ca_‐ and IK_Ca_-mediated EDH hyperpolarizes VEC, K^+^ efflux could stimulate NKA in vascular smooth muscle cells (VSMCs; Garland and Dora, 2017). To test if NKA is involved in CPA-induced vasorelaxation, we applied ouabain (100 μM, NE-vasoconstriction value 7.84 ± 1.55 mN) to inhibit NKA in the presence of L-NNA and INDO. CPA-induced CRC and R_max_ (51.45 ± 6.90%), were significantly attenuated by ouabain (*p* < 0.0001, Figure 5A). Furthermore, when extracellular K^+^ was omitted (0 K^+^, NE-vasoconstriction value 8.36 ± 1.95 mN) to silence NKA, CPA-induced CRC and R_max_ (41.56 ± 8.15%) were also significantly attenuated (*p* < 0.0001, Figure 5B). Since both ouabain and 0 K^+^ significantly reduced CPA-induced R_max_ (Figures 5A,B), we concluded that CPA-induced EDH activated NKA, leading to the vasorelaxation of mesenteric arteries.

### Na^+^/Ca^2+^ Exchanger in the SOCE/EDH-Mediated Vasorelaxation

**Figure 6:**
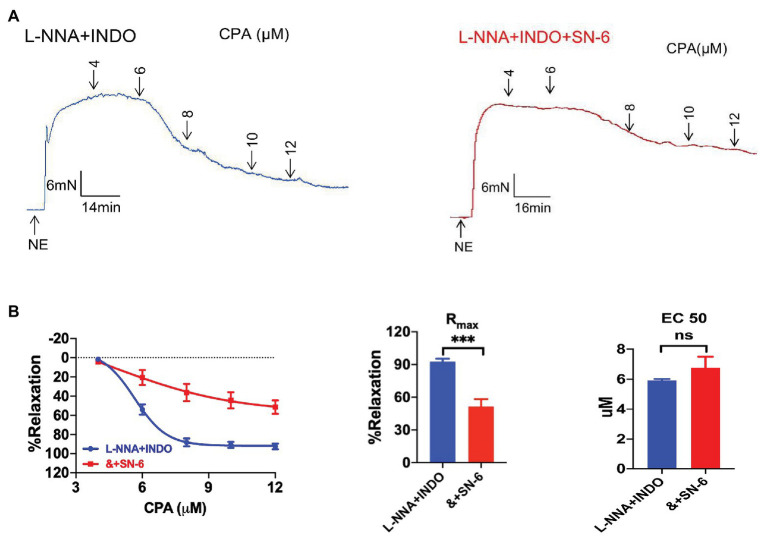
The involvement of sodium-calcium exchanger in the EDH-mediated vasorelaxation of mesenteric arteries.**(A)** Representative tracings of CPA-induced dose-dependent vasorelaxation in the presence of either L-NNA + INDO (the left panel) or L-NNA + INDO + 10 μM SN-6 (the right panel). **(B)** Summary data showing the CRC, R_max_, and EC_50_ of CPA-induced vasorelaxation in the presence of either L-NNA + INDO (*n* = 6) or L-NNA + INDO (&) + 10 μM SN-6 (*n* = 6). Data were expressed as percentage of NE (5 μM)-induced vasoconstriction and shown as means ± SEM. ^***^p < 0.001 and ns, no significance.

Endothelium-dependent hyperpolarization signal is mediated not only by Ca^2+^-activated IK_Ca_ and SK_Ca_ channels in VEC, but also by NKA and Na^+^/Ca^2+^ exchanger (NCX) in VSMCs (Cocks et al., 1988), in which they play a critical role in regulating vascular tone (Matchkov et al., 2007). Therefore, we applied SN-6 (10 μM, a selective inhibitor of Na^+^/Ca^2+^ exchanger, NE-vasoconstriction value 8.44 ± 1.65 mN) in the presence of L-NNA and INDO, to test whether NCX is also involved in CPA-induced vasorelaxation. CPA-induced CRC and R_max_ (51.45 ± 6.90%) were significantly inhibited (Figures 6A,B), indicating that NCX plays a critical role in CPA-induced EDH-mediated vasorelaxation.

### Successfully Created a Mouse Colitis Model

**Figure 7:**
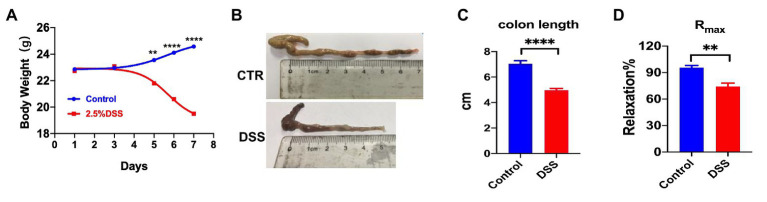
Comparison of body weight and colon length between control mice and dextran sulfate sodium (DSS)-induced colitis of mouse model. **(A)** Summary data showing the time courses of body weight in control mice (*n* = 6) or colitis mice treated with 2.5% DSS (po) for 7 days (*n* = 6). **(B)** Representative photos of colon length in control and colitis mice. **(C)** Summary data showing colon length in control mice (*n* = 6) and colitis mice (*n* = 6). **(D)** Summary data showing the acetylcholine (ACh)-induced (10 nM–1 mM) maximal vasorelaxation from normal mice and colitis mice (*n* = 4). Data were shown as means ± SEM. ^**^p < 0.01, ^****^p < 0.0001.

Although in IBD patients, blood flow to chronically inflamed regions of gut was reduced (Hatoum et al., 2003a; Hatoum and Binion, 2005), it is not known if SOCE/EDH signals in the mesenteric circulation are involved in the pathogenesis. We first created DSS-induced colitis in a mouse model and found that the body weight and colon length of colitis mice were significantly reduced (Figures 7A,C). After 4 days of ingestion of water containing 2.5% DSS, the mice had bloody stools and their body weight was significantly lower than that of the control group after a week. On day 7, the mice were sacrificed through cervical dislocation, and the intestinal segment from the anus to the ileocecal area was removed. There was considerable bleeding in the intestinal segment as well as a significant reduction in the colon length of colitis mice (Figures 7B,C). To verify the dysfunctions of vascular endothelial cells in colitis, we compared the ACh-induced (10 nM–1 mM) relaxation between normal mice and DSS-induced colitis mice (R_max_: 95.65% vs. 74.18%, *p* < 0.05, Figure 7D). In summary, the DSS-induced colitis in a mouse model was successful.

### The Vasorelaxation Through SOCE/EDH Was Impaired in Colitis

**Figure 8:**
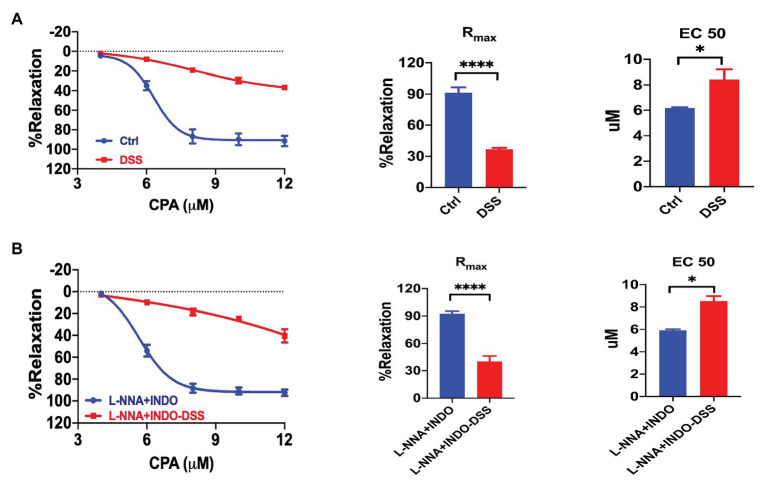
Impairments of the SOCE/EDH-mediated vasorelaxation in colitis. **(A)** Summary data showing the CRC, R_max_, and EC_50_ of CPA-induced vasorelaxation in control mice (*n* = 6) or colitis mice treated with 2.5% DSS (po) for 7 days (*n* = 6). **(B)** Summary data showing the CRC, R_max_, and EC_50_ of CPA-induced vasorelaxation as described in **(A)** but in the presence of L-NNA + INDO (*n* = 6). Data were expressed as percentage of NE (5 μM)-induced vasoconstriction and shown as means ± SEM. ^*^p < 0.05, ^****^p < 0.0001.

We compared the CPA-induced vasorelaxation between the control and colitis mice. The CRC in response to CPA treatment was markedly impaired in colitis mice (Figure 8A). The R_max_ (36.71 ± 1.40%) in colitis mice were significantly reduced compared to that in control mice (R_max_ 91.46 ± 5.09%, *p* < 0.0001). The EC_50_ of CPA-induced vasorelaxation was higher in colitis mice (EC_50_ 8.43 ± 0.78 μM) compared to that in control mice (EC_50_ 6.16 ± 0.07 μM, *p* < 0.05). Therefore, the CPA/SOCE-mediated endothelium-dependent vasorelaxation is impaired in the pathogenesis of colitis.

To further understand the contribution of EDH, we compared the CPA-induced vasorelaxation between control and colitis mice in the presence of a combination of L-NNA and INDO. After inhibition of NO plus PGI_2_ using L-NNA and INDO, CPA-induced EDH-mediated vasorelaxation was largely impaired in colitis mice (Figure 8B). Similarly, the R_max_ (40.44 ± 5.91%) in colitis mice were significantly reduced compared to that in control mice (R_max_ 92.55 ± 2.79%, *p* < 0.0001). The EC_50_ of CPA-induced vasorelaxation was higher in colitis mice (EC_50_ 8.54 ± 0.44 μM) compared to that in control mice (EC_50_ 5.92 ± 0.10 μM, *p* < 0.05). Therefore, CPA/SOCE/EDH-mediated vasorelaxation is largely impaired in the pathogenesis of colitis.

## Discussion

The main findings of this study are as follows: (1) CPA-induced depletion of ER Ca^2+^ induces an endothelium-dependent dilation that requires activation of SOCE; (2) this vasorelaxation upon CPA-induced depletion of ER Ca^2+^ mainly relies on EDH; (3) both NKA and NCX are involved in the vasorelaxation through the CPA/SOCE/EDH mechanism; and (4) the CPA/SOCE/EDH-mediated vasorelaxation is defective in colitis.

[Ca^2+^]_i_ plays a critical role in regulating vasoconstriction and vasorelaxation (Rocha and Bendhack, 2009), as an important second cell messenger. In the resting state, the intracellular and extracellular Ca^2+^ levels remain relatively stable, and the extracellular Ca^2+^ is much higher than the intracellular Ca^2+^. The fine regulation of [Ca^2+^]_i_ is of great significance for maintaining the normal function of endothelial cells and VSMCs (Garland et al., 2017). In molecular pathway of the SOCE, STIM protein senses the depletion of Ca^2+^ in the endoplasmic reticulum, and induces Ca^2+^ influx through coupling with protein Orai (Liou et al., 2005; Zhang et al., 2006). In non-excitable cells, such as vascular endothelial cells, the SOCE plays an important role in regulating cellular Ca^2+^ balance (Várnai et al., 2009). Under physiological conditions, IP_3_ is the usual stimulus for the ER/Ca^2+^ release *via* IP_3_ receptors which results in the loss of Ca^2+^ from the ER, leading to SOCE activation; and thereby the degree of SOCE activation is related to the ER/Ca^2+^ depletion degree (Taylor, 2006).

As a selective SERCA inhibitor, CPA inhibits Ca^2+^ uptake into the ER to finally activate SOCE so that it is often used as an SOCE activator. In the present study, we found that CPA indeed activated endothelial SOCE through depletion of the ER Ca^2+^, indicating it is a reliable SOCE activator. Afterwards, we applied two selective SOCE blockers and an Orai blocker to inhibit the SOCE/Orai channels, and revealed that CPA-induced vasorelaxation was significantly inhibited, proving that CPA exerts endothelium-dependent vasorelaxation likely through the SOCE/Orai channels.

The mesenteric artery is a recognized resistance vessel model and plays an important role in regulating blood flow to the intestine and in maintaining blood pressure. When the resistance vessels relax, blood flow to the organ increases and vice versa. NO and EDH derived from endothelial cells have been recognized as the main factors regulating vasorelaxation (Félétou and Vanhoutte, 2007). NO activates guanylate cyclase on VSMC to increase intracellular cGMP, which exerts a vasorelaxation effect (Vanhoutte et al., 2017). PGI_2_ activates receptors on VSMC, causing vasorelaxation (Parkington et al., 2004), and EDH is known as a non-NO and non-PGI_2_ endothelium-dependent hyperpolarization (Busse et al., 2002; Matoba and Shimokawa, 2003). Under physiological conditions, both NO and EDH are the major vasodilators: the former is dominant in conduit arteries, but the latter is critical in resistance vessels (Shimokawa et al., 1996). Importantly, endothelial dysfunction leads to reduced generation of NO, which in turn stimulates EDH, as a compensatory mechanism to maintain the endothelium-dependent vasorelaxation of resistance vessels (Ueda et al., 2005; Yada et al., 2018), highlighting the critical role of EDH in resistance vessels.

Although the nature of EDH is still elusive so far (Félétou and Vanhoutte, 2007), it has been generally accepted that it is the Ca^2+^ increase in endothelial cells that activates IK_Ca_ and SK_Ca_ channels to induce membrane hyperpolarization (Gluais et al., 2005). In this study, we revealed that SOCE activation upon the ER/Ca^2+^ store depletion by CPA initiates relaxation of mesenteric artery that is mediated by EDH, which is supported by the following evidence: (1) mechanical removal of endothelium resulted in nearly complete inhibition of CPA-induced vasorelaxation; (2) high potassium to pre-contract the vessels significantly inhibited CPA-induced vasorelaxation; (3) while both NO and PGI_2_ inhibitors did not alter the CPA-induced vasorelaxation, K_Ca_ inhibitor significantly inhibited it but K_Ca_ activator promoted it; and (4) the CPA-induced vasorelaxation was attenuated by SOCE blockers, and the endothelial SOCE was confirmed by single cell Ca^2+^ imaging. Although all inhibitors applied in the present study will affect both vascular endothelial and smooth muscle cells, the SOCE function is opposite: it induces endothelium-dependent vasorelaxation as shown in our study, but it enhances intracellular calcium level in smooth muscle cells to induce vasoconstriction. Our findings are consistent with the reports in different animal arteries from other laboratories (Dora et al., 2003; Crane and Garland, 2004; Edwards et al., 2008), and electrophysiological experiments confirmed CPA-induced hyperpolarization in rat mesenteric arteries (Fukao et al., 1995), further supporting our notion of CPA-induced endothelial SOCE/EDH mechanism.

**Figure 9:**
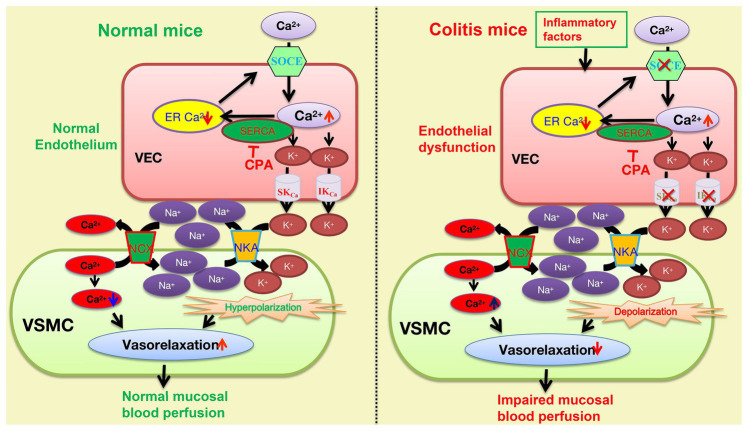
The underlying mechanisms of the SOCE/EDH-mediated vasorelaxation of mesenteric arteries in health and its impairments in the pathogenesis of colitis. The SOCE/EDH-mediated vasorelaxation in health (left panel) and its impairments in colitis (right panel). CPA inhibits the SERCA to activate the SOCE and induce Ca^2+^ signaling that stimulates IK_Ca_ and SK_Ca_ on vascular endothelial cells (VEC), leading to K^+^ efflux. An increase in extracellular K^+^ between VEC and VSMC activates NKA to eventually cause vasorelaxation through hyperpolarization. Moreover, NKA activation reduces [Na^+^]_i_ in VSMC, which stimulates Na^+^/Ca^2+^-exchange (NCX) activity to decreases [Ca^2+^]_i_, resulting in further vasorelaxation. However, the SOCE/EDH-mediated vasorelaxation is likely impaired by inflammatory factors-induced endothelial dysfunction in the pathogenesis of colitis. SOCE, store operated Ca^2+^ entry; VEC, vascular endothelial cells; VSMC, vascular smooth muscle cells; SERCA, sarcoendoplasmic reticulum calcium transport ATPase, IK_Ca_ and SK_Ca_: intermediate and small conductance of Ca^2+^-activated K^+^ channels; NKA, Na^+^/K^+^-ATPase; and NCX, Na^+^/Ca^2+^-exchanger.

Although both NKA and NCX are known to jointly participate in the vasorelaxation mechanism of EDH signals (Cocks et al., 1988; Garland and Dora, 2017), if they are involved in the SOCE/EDH mechanism is still elusive. After applying either ouabain or 0 K^+^ solutions to inhibit NKA and SN-6 to inhibit NCX, we observed that CPA-induced vasorelaxation was significantly inhibited by each of them, suggesting their involvement. Therefore, our results indicated that CPA activates endothelial SOCE to raise [Ca^2+^]_i_ that stimulates the IK_Ca_ and SK_Ca_. The efflux of K^+^ results in accumulation between endothelial and smooth muscle cells, which leads to NKA activation and hyperpolarization to inactivate the voltage-dependent calcium channels, eventually resulting in vasorelaxation. Concurrently, NKA activation decreases [Na^+^]_i_ in smooth muscle cells, which in turn stimulates NCX activity to decrease [Ca^+^]_i_ (Guo et al., 2020), further enhancing vasorelaxation (Figure 9).

The intestinal blood circulation plays an important role to maintain normal GI function (Gasbarrini et al., 2008), and mesenteric artery is critically involved in blood perfusion in the intestinal mucosa. It was previously reported that dysfunction of intestinal microvasculature impaired mucosal wound healing, which may lead to refractory mucosal ulceration (Papa et al., 2008). Although loss of NO generation and change in PGI_2_-dependent vasorelaxation (Hatoum et al., 2003b) resulted in dysfunction of the microvascular relaxation in colitis (Mori et al., 2005), it has not been addressed if the CPA-mediated vasorelaxation is impaired in colitis. By systematically compared the vasorelaxation of the mesenteric arteries in healthy and colitis mice in terms of the CPA/EDH mechanism, we revealed that this pathway was severely impaired in colitis. This may lead to the reduced blood perfusion to the intestinal mucosa, which will affect mucosal repair after injury to finally promote the progression of colitis.

## Data Availability Statement

The raw data supporting the conclusions of this article will be made available by the authors, without undue reservation, to any qualified researcher.

## Ethics Statement

The animal study was reviewed and approved by the Animal Ethical Committee of Chongqing Medical University and the Guide for the Care and Use of Laboratory Animals published by the US National Institutes of Health.

## Author Contributions

HD conceived the study, designed most experiments, and wrote and finalized the manuscript. LZ performed most experiments and data analysis. FX designed and XC performed some experiments. All authors contributed to the article and approved the submitted version.

### Conflict of Interest

The authors declare that the research was conducted in the absence of any commercial or financial relationships that could be construed as a potential conflict of interest.
